# Collectanea Medica

**Published:** 1813-01

**Authors:** 


### COLLECTANEA MEDICA,

CONSISTING OF

ANECDOTES, FACTS, EXTRACTS, ILLUSTRATIONS, QUERIES, SUGGESTIONS, &c.

RELATING TO THE

*History or the Art of Medicine, and the Auxiliary Sciences.*

---

### *Has the Physical Condition of the Head any immediate influence on that of the Mind?*

PROFESSOR PINEL has remarked, "I am cautious how I decide; and confine myself to mark the line which separates truth from probability. The varieties of form, the exact determination of measures, and the relative proportions tions of the parts, are the only subjects which I profess to discuss. The rest I leave to the wild field of conjecture, which, in other words, is a species of vesania, common enough in the world, but which has not yet been recognised at the *Petites Maisons*.* The anatomy and pathology of the brain are yet involved in extreme obscurity. Greding dissected two hundred and sixteen maniacal subjects, and he details all the peculiarities which he observed in the meninges, the substance of the brain, the ventricles, the pineal gland, and the cerebellum. But, as those maniacs died by disorders unconnected with their mental ailments, we can form no just conclusions from the morbid appearances which presented themselves. Many varieties of structure might likewise accidentally co-exist with the lesions of the mental function, without having any immediate connection with them. The same may be said of the experiments of a similar nature, by Haslam in England, and Chiaruggi in Italy. I have attended at thirty-six dissections in the Hospital de Bicetre, and I can declare that I have never met with any other appearances within the cavity of the cranium, than are observable on opening the bodies of persons who have died of apoplexy, epilepsy, nervous fevers, and convulsions. From such data, what light can be thrown on the subject of insanity? In one of my dissections, indeed, I recollect to have found a steatomatous tumor, about the size of a pullet's egg, in the middle of the right lobe of the brain; but the disease, in that instance, was not insanity but hemiplegia. What a field would have been opened for hypothesis and comment, had this person been likewise afflicted with insanity! What an additional motive for circumspection and reserve in deciding the physical causes of mental alienation!"

---

*Rules for Diet, from Suvorof's Catechism, or "Discourse under the Trigger."*

"Have a dread of the hospital! German physic stinks from afar, is good for nothing, and rather hurtful: a Russian soldier is not used to it. Messmates know where to find roots, herbs, and pismires. A soldier is inestimable. Take care of your health! Scour the stomach when it is foul! Hunger is the best medicine! He who neglects his men, if an officer, *arrest*—if a sub-officer, *sticks*—and to the private, *lashes*, if he neglects himself. If loose bowels want food, at sun-set a little gruel and bread. For costive bowels, some purging plant in warm water, or the liquorice root. Re-

---

* An institution at Paris for the reception of insane persons.

member, gentlemen, the *field physic of Dr. Bellipotski!** In hot fevers eat nothing, even for twelve days, and drink your soldier's quas†—that's a soldier's physic. In intermitting fevers, neither eat nor drink. It 's only a punishment for neglect, if health ensues. In hospitals, the first day the bed seems soft—the second, comes French soup—and the third, the brother is laid in his coffin, and they draw him away! One dies, and ten companions around him inhale his expiring breath. In camp, the sick and feeble are kept in huts, and not in villages; there the air is purer. Even without an hospital, you must not stint your money for medicine, if it can be bought; nor even for other necessaries. But all this is frivolous—we know how to preserve ourselves! Where one dies in an hundred with others, we lose not one in five hundred in the course of a month. For the healthy, *drink, air,* and *food*—for the sick, *air, drink,* and *food!!*

* Supposed to be a manual of medicine, published for the use of the army.

† A sour beverage, made of fermented flour and water.

